# Clinically relevant evaluation of the antimicrobial and anti‐inflammatory properties of nanocrystalline and nanomolecular silver

**DOI:** 10.1111/wrr.13249

**Published:** 2024-12-20

**Authors:** Matthew W. Pletts, Robert E. Burrell

**Affiliations:** ^1^ Department of Biomedical Engineering University of Alberta, Research Transition Facility Edmonton Alberta Canada; ^2^ Department of Biomedical Engineering University of Alberta Canada

**Keywords:** anti‐inflammatory, antimicrobial, burn, clinical, dressing, mechanism, medicine, nanocrystalline, properties, silver, structure, wound

## Abstract

Burns and chronic wounds present significant challenges in wound management due to risks of infection, excessive inflammation, and prolonged healing. Silver‐based treatments have long been central to burn care, but limitations have prompted the exploration of nanocrystalline silver as an alternative, with its nanoscale properties offering distinct benefits. This paper reviews the structure, properties, mechanisms of action, and clinical applications of nanocrystalline silver in burn and general wound management, with particular emphasis on how wound healing processes inform the application of these dressings. Nanocrystalline silver's high surface area‐to‐volume ratio and crystal structure enhance its antimicrobial and anti‐inflammatory efficacy. Nanocrystalline silver's mechanisms of action are disrupting cellular functions, inducing DNA damage, and inhibiting biofilms. Clinical studies demonstrate accelerated healing and reduced inflammation compared to traditional treatments. Whilst nanocrystalline silver dressings are costly, their effectiveness in lowering drug‐resistant infections and minimising complications supports a financial case for their use, potentially reducing overall wound care expenses. Considerations of cytotoxicity, allergic reactions, and accessibility underscore the importance of individualised treatment selection based on wound and patient factors. In conclusion, nanocrystalline silver holds substantial promise in burn wound management, and further research is warranted to optimise its therapeutic potential and economic benefits in clinical practice.

**Abbreviations**
AgNPs: ultra‐small silver nanoparticles
BCC: body‐centred‐cubic
FCC: face‐centred‐cubic
HCP: hexagonal close‐packed
HRTEM: high‐resolution transmission electron microscopy
IL‐1β: interleukin‐1β
IL‐6: interleukin‐6
MMP: matrix metalloproteinase
PAMPS: pathogen‐associated molecular patterns
PRRs: pattern‐recognition receptors
SEM: scanning electron microscopy
SSD: silver sulphadiazine
TEM: transmission electron microscopy
TLRs: toll‐like receptors
TNF‐α: tumour necrosis factor‐α
XPS: X‐ray photoelectron spectroscopy

## 1 INTRODUCTION

Burns have been described as the “universal trauma model,” given that burns demonstrate many of the body's innate responses to injury, inflammation, and infection.^1^ Treating burns throughout history primarily relied on an Aristotelian approach until the onset of the First World War.^2^ However, wounds endured during trench warfare, often contaminated by field conditions, resulted in a high mortality rate in patients due to necrotizing wound infections.^3^ The unprecedented morbidity of necrotizing burn/wound infections resulted in existing treatment protocols being questioned and new ones being developed.^2^ One of the standard treatments for burns that emerged during the First World War was the topical application of tannic acid; however, it was observed that tannic acid delayed the onset of infection.^4^ Recently, interest has been resurgent regarding an antimicrobial used since ancient times, honey.^2^ However, a variety of the antimicrobial properties of honey, as well as studies of honey for low‐quality burn treatments, have resulted in the treatment modality not being widely used.^5^ Modern methods to prevent wound infection and control for excessive inflammation that is not silver‐based include topical antimicrobial agents, negative pressure wound therapy, biological dressings, and pharmacological interventions. The treatment choice depends on the specific type and severity of the wound or burn. Silver‐based treatments, particularly nanocrystalline silver, have become a central focus in modern burn care due to their potent antimicrobial and anti‐inflammatory effects, making them an essential option in managing complex burn wounds.

## 2 MATERIALS AND METHODS

A comprehensive literature search was conducted to identify relevant studies on nanocrystalline silver dressings. The following databases were searched: PubMed, Scopus, Web of Science, and Cochrane Library. The search was performed using the following keywords and their combinations: “nanocrystalline, silver”, “antimicrobial”, “anti‐inflammatory”, “mechanism”, “properties”, “structure”, “clinical”, “wound”, “burn”, “dressing”, “medicine.” The search was limited to articles published in English. Studies were included if they investigated the properties, mechanisms of action, or clinical efficacy of nanocrystalline silver dressings and if they were original research articles, systematic reviews, or meta‐analyses. Studies were excluded if the methodology and materials used were not sufficiently clear.

## 3 DISCUSSION

### 3.1 Historical uses of silver

Silver has been used in medical treatments since 4000 B.C.E.^6^ Throughout the 1800s, silver nitrate was used to treat “ulcerations and infected wounds and prevent gonococcal ophthalmic infections in newborns”.^7^ The use of silver solutions as a treatment of burns diminished until interest was revived by the 1965 article by Moyer et al., “Treatment of large human burns with 0.5% silver nitrate solution”.^8^ Silver nitrate leads to minimal insensitivity or bacterial resistance.^8^ A complication of silver nitrate use is a decrease in serum chlorine and sodium concentrations due to ion exchange between Ag^+^ and protein, Cl^−^, CO_3_^−^, and HCO_3_^−^.^8^ The general assumption is that silver is not widely taken into the systemic circuit following topical application. However, the post‐mortem examination of two patients treated with silver nitrate revealed elevated concentrations of silver within the kidneys, liver, spleen, and muscles. Silver concentrations observed in the two patients ranged from 0.14 to 2.0 parts per million (ppm), compared to a control of 0.025 ppm.^9^ The efficacy of 0.5% silver nitrate solution was investigated in a treatment group of 100 patients who had burns covering, on average, 53% of the body and had been treated with 0.5% silver nitrate. The conclusion was that the burns were predisposed to invasive infection after treatment with 0.5% silver nitrate, even when silver nitrate solution treatment was initiated soon after injury.^10^ Infection of burns following topical application of silver nitrate was minimal in burns covering less than 60% of the body. Mortality from deeper burns covering greater than 60% of the body was primarily caused by sepsis following invasive infection, primarily by *Pseudomonas aeruginosa*.^10^ Silver nitrate treatment has decreased mortality, complications, and hospitalisation.^11^ However, there is the argument that this observation is not due primarily to the use of silver nitrate but rather the regular changing of dressings and the cleanliness required by the treatment regime.^12^ Another silver solution used for the treatment of burns is silver sulphadiazine. A survey of topical treatments used in American burn centres showed that a treatment regime utilising silver sulphadiazine is the most common approach.^13^ A standard treatment, alternating agents, alternates the topical application of silver sulphadiazine (SSD) and mafenide every 12 h.^2^ For unexcised wounds, in the military setting, the alternating agent treatment approach is, at present, the recommended treatment option.^14^ For more extensive burns, total body excision, followed by closure via autograft or a combination of autograft and allograft autograft, is used.^15^ However, regarding this surgical approach, not all patients are suitable candidates. Inequalities in healthcare coverage and mass‐casualty scenarios limit the feasibility of the surgical approach as a primary treatment option.^14^ In a study of serum and urine concentrations of silver following the application of silver sulphadiazine on burn patients, elevated silver secretion was observed. The secreted and serum concentrations of silver remained elevated throughout the period of hospitalisation.^16^ Following topical application of silver sulphadiazine, it is estimated that approximately 10% of the SSD is excreted via the kidneys.^17^ Leukopenia is observed temporarily during the SSD treatment regime in some patients, onsetting usually 2–3 days following the commencement of treatment and disappearing with the long‐term application.^18^ Modern applications of SSD go by the trade names Silvadene™, SSD AF™, Thermazene™, and Flamazine™.

### 3.2 Nanocrystalline silver structure and properties

When describing the structure of nanocrystalline silver materials, it is necessary to discuss the terminology used. Within the literature, there is no consistent agreement on the definitions of terms, including particle, crystal, and nanocrystalline. For this investigation, working definitions of the terms will be provided here to improve clarity during later discussions.

A silver particle will be defined as a face‐centred cubic unit consisting of 14 atoms of silver. Therefore, any number of silver particles will be multiples of 14 atoms. When referring to colloidal silver or non‐nanocrystalline silver dressings, colloidal silver may be referred to as silver nanomolecular solutions or deposited substrates. Nanocrystalline materials may be single‐phase or multiphase polycrystals with a crystal size typically ranging between 1 and 10 nm.^19^ Nanocrystalline materials have high crystal‐to‐grain boundary volume ratios (typically one‐to‐one). When describing the atomic arrangement of materials, they are typically described as amorphous/glasslike or crystalline. Crystals and glasses differ in their presence or absence of long‐ or short‐range order. Nanocrystalline materials utilise atomic arrangements in the cores of lattice defects to form solids with novel atomic structures. Due to the contained atomic arrangement of adjacent lattices, the atoms within the core differ from unconstrained arrangements in amorphous or crystalline materials.

Nanocrystalline silver consists of silver crystals within a nanoscale size range of approximately 10–20 nm. Silver at this scale has unique properties and characteristics compared to bulk silver. Unique characteristics of nanocrystalline silver include a high crystal surface area‐to‐volume ratio and different crystal structures compared to macro silver. The face‐centred‐cubic (FCC) structure is silver's most commonly observed crystal structure.^20^ However, there can be various crystal structures of silver at the nanoscale, including FCC, body‐centred‐cubic (BCC), or hexagonal close‐packed (HCP).^21^ The transition from FCC to HCP is solvent‐dependent and attributed to the specific stacking process. Conversely, the formation of BCC structures is solvent‐independent and attributed to van der Waals attractions. Additionally, the deposition temperature and chain length of the coating agent determine the transition from FCC or HCP to BCC. The variation of the crystal structure is dependent on the conditions of synthesis. Silver nanostructure characteristics result in unique dissolution properties.^22^ This included the release of Ag^0^ clusters, Ag^+^ ions, and Ag^+++^ ions when they were dissolved.^23^ Additionally, in biologically active films, XPS imaging has detected oxygen species in various chemical states, which are found in various quantities.^24^

By decreasing the size of crystals, the crystal surface area increases relative to the volume of the silver. This property facilitates greater interaction with the wound environment and promotes the release of silver clusters and silver ions. An increase in the total fraction of silver atoms located within the grain boundaries and particle surfaces may facilitate nanocrystalline silver's antimicrobial and anti‐inflammatory properties.^22^ The surface morphology of silver dressings has been explored in films by adjusting gas concentrations, power levels, and other factors.^24^ In a film developed with 4% oxygen and 96% argon plasma gas at 0.3 kW, a 900 nm thick film revealed the presence of large columnar structures that are polycrystalline, with triangular cross‐sections. This differentiates from the single particle size observed at other design conditions. Nanocrystalline silver also features grain faceting and the presence of surface crystallites. Increasing power during deposition results in a surface morphology featuring spherical particles and approximately equi‐sized grain boundaries of 15 nm. Biologically active silver films are very porous even though some crystallites are agglomerated. Interestingly, nanocrystalline grains on the surface of columnar grains occur in biologically active films developed under 96%Ar–4%O_2_ or 99%Ar–1%O_2_. The grain boundaries between the larger columnar grains and the nanocrystalline grain appear more important than the porosity of biologically active films.

Silver ions are readily precipitated as silver chloride or silver proteinate; both precipitates have very limited antimicrobial activity.^25^ Due to the precipitation of silver chloride, following the application of silver nitrate to a wound, the silver cannot penetrate deeper tissue layers where infection is already established. Additionally, there is concern about silver nitrate‐induced electrolyte imbalance. In this treatment regime, chloride blood levels decrease as the anion binds to silver and precipitates the non‐soluble salt, silver chloride. There are also major decreases in sodium and lesser decreases in potassium. Whilst sodium is lost, intracellular potassium is released into the extracellular environment to maintain iso‐osmolarity. This results in potassium serum levels that may not reflect normal intracellular potassium levels. The risk for developing a hyponatremic (abnormally low physiological sodium concentration) and/or hypochloremic (abnormally low physiological sodium concentration) state is higher when using silver nitrate, colloidal silver, and/or silver sulphadiazine compared to nanocrystalline silver. This is because of the difference in concentrations and species of silver released. Silver nitrate releases silver in concentrations as high as 3176 ppm (0.5% silver nitrate). Silver sulphadiazine releases silver in concentrations as high as 3000 ppm (1% silver sulphadiazine) but reaches equilibrium at 90 ppm. The silver ions released by silver nitrate and silver sulphadiazine are mostly inactivated due to reacting with anions in the wound environment. This suggested that replenishing Ag^+^ ions by multiple applications of silver nitrate provides an antimicrobial activity that is overcome by chloride ions that bind and precipitate the silver ions.^26^ Nanocrystalline silver facilitates bactericidal properties at lower concentrations of silver due to multiple forms of silver being released. Nanocrystalline silver dressings release Ag^0^, Ag^+^, and higher oxidation states of silver that are less readily deactivated by chloride or other organic matter in the wound. Due to the multiple silver species, nanocrystalline silver dressings are effective whilst releasing silver at concentrations of approximately 70 ppm.^27^

The crystal structure of nanocrystalline silver differs from bulk silver. As a result of smaller crystal sizes, there is a higher density of crystal defects, including grain boundaries. Grain boundaries exist in regions where adjacent crystal grains or crystals meet and can influence the material's properties. In the case of nanocrystalline silver dressings, these grain boundaries contribute to increased reactivity, enhancing the dressing's antimicrobial properties. High‐resolution transmission electron microscopy (HRTEM) has shown that biologically active nanocrystalline silver has grain boundaries in the order of 15 nm and a high density of growth twins whose incoherent twin boundaries intersect grain boundaries.^24^ Additionally, temperature affects grain boundaries and the biological activity of nanocrystalline silver. Annealing is a process that decreases defects such as dislocations and interstitials in a material. Annealing dressings above 75°C increase grain growth and decrease biological activity.

Nanocrystalline silver includes a high crystal surface area‐to‐volume ratio and different crystal structures compared to macro silver. These features of nanocrystalline silver contribute to enhancing antimicrobial and anti‐inflammatory effects.

Recent advancements in silver nanoparticle technology are refining particle size and delivery methods to enhance antimicrobial effectiveness whilst reducing toxicity concerns. A direction currently under investigation is utilising ultra‐small silver nanoparticles (AgNPs) under 3 nm, which demonstrate improved biofilm penetration compared to larger particles and require lower concentrations to achieve an antimicrobial effect.^28^ However, these nanoparticles often rapidly oxidate due to their high reactivity and instability, limiting their efficacy in clinical settings. To address this, researchers have developed biocompatible hydrogels impregnated with ultra‐small AgNPs, which stabilise the nanoparticles and provide a sustained‐release mechanism that maintains antibacterial potency over extended periods. These hydrogels serve as reservoirs, releasing silver gradually to minimise toxic effects on human cells whilst demonstrating significant biofilm eradication, up to 80% of biofilms in single‐ and multi‐species models. Another emerging advancement utilises stimuli‐responsive hydrogels loaded with AgNPs, emerging as a dynamic solution designed to release silver ions in response to wound pH or temperature changes.^29^ This dual‐responsive hydrogel system remains inactive at an acidic pH, which is common in non‐infected wounds. Still, it becomes highly active in the alkaline conditions associated with infection, releasing over 90% of the silver ions to eliminate pathogens. The targeted release approach reduces the risk of adverse side effects and improves antibacterial efficiency by aligning treatment with the wound's physiological needs. These innovations in AgNP delivery hold the potential for enhancing wound management by providing targeted, long‐lasting antimicrobial action.

### 3.3 Mechanisms of action

This section discusses the multiple mechanisms of action regarding nanocrystalline silver's antimicrobial and anti‐inflammatory properties. These mechanisms of action have been supported by discussing details of the antimicrobial and inflammatory pathways, the restoration of homeostasis, and how excessive inflammation results in decreased wound healing.

Silver acts as an antimicrobial agent via multiple mechanisms, interrupting enzymes and components associated with cellular respiration, impairing DNA functions, altering cellular membrane permeability, and disrupting biofilms.^30^ Silver ions disrupt metabolic pathways within cells and lead to the overproduction of hydroxyl radicals (a reactive oxygen species), leading to cell death by damaging cellular components within the microorganism.^31^ Like other heavy metals, silver poisons components of the microbial electron transport chain, which impedes the microorganism's cellular respiration.^30^ The interaction of silver on DNA has been studied principally using radioactive silver isotopes, ^110^Ag. The interaction between silver ions and DNA occurs because silver binds with DNA with 10 times the affinity than cellular proteins and polysaccharides and binds to DNA with 40 times the affinity than RNA.^32^ The denaturation of DNA because of silver occurs between the silver ion and two disulphide bonds.^33^ Silver nanomoleculars act as antiviral agents against HIV‐1, hepatitis B virus, monkeypox virus, herpes simplex virus type 1, and respiratory syncytial virus. Silver acts as an antiviral against HIV‐1 by denaturing disulphide bonds in the carboxyl half of the gp120 domain. This inhibits the binding of gp120 to a CD4 receptor on a host cell.^34^ Additionally, during the inhibitory period of a bacterial growth cycle, there is an increase in the amount of silver bound to DNA, and the growth does not initiate until the level of bound silver decreases. This suggests that inhibition of bacterial growth occurs when many base pairs throughout the DNA helix are bound to silver; it is also possible that DNA segments attached to silver are selectively removed during the resynthesis of the excised region. Another mechanism by which silver acts as an antimicrobial agent is by interacting with the outer membrane of bacteria. This interaction results in the degradation and death of the microorganism.^35^ Using TEM and SEM, it has been observed that *E. coli* cells are damaged when treated with silver nanomolecular; within the cell wall, silver results in “pitting” to the tightly packed lipopolysaccharide molecules, which provide a permeable barrier. Furthermore, morphological changes occur to the cell envelope, further increasing permeability. Silver accumulates in the bacterial membrane, increasing permeability and cell death.^36^ This results because the treated cells cannot regulate transport through the plasma membrane. Biofilms are an assembly of surface‐associated microbial cells encapsulated in an extracellular matrix comprising a polymeric substance.^37^ This assembly of microbial cells is not removed by gentle rinsing (described as irreversibly associated) with a surface. Organisms within biofilms exhibit differences to feely suspended planktonic organisms, primarily by transcribing different genes, improving nutrient and water supply, and improving lateral gene transfer. Biofilms can form on many surfaces, including living tissue or medical devices. Biofilms offer protection from adverse conditions and can act as reservoirs for pathogenic organisms.^38^ Given the characteristics of biofilms, they can be responsible for various nosocomial infections, such as catheters and wound dressings. Treatments of infections following the formation of biofilms are often challenging with existing agents, and often, the only remedy is the physical removal of the biofilm (by removing the medical device it is attached to). Silver nanomoleculars effectively inhibit the formation of biofilms by approximately equal gram‐negative and gram‐positive microorganisms.^39^ Furthermore, silver nanomoleculars prevent biofilms from forming and are lethal to the bacteria within the film.^38^ By utilising multiple mechanisms of action, medical applications of silver rarely result in resistance. Furthermore, cross‐resistance between antibiotics and silver is rare.^30^

Table 1 tabulates the mechanisms of action of nanocrystalline silver dressings that contribute to wound healing. Inflammation is an essential homeostatic response to tissue trauma because it destroys toxic agents at the injury site by promoting blood vessel vasodilation and restoring tissue homeostasis. Cutaneous wound healing involves an inflammatory reaction, cell proliferation, and remodelling.^40^ The inflammatory phase initiates with the coagulation of blood from the lesion; this contributes to the maintenance of its integrity. Coagulation occurs due to the interaction of thrombocytes and platelets within a fibrin network. The resulting barrier protects against infection and helps to restore homeostasis. Inflammation is onset with an influx of leukocytes to the wounded area. The presence of oedema and erythema characterises this inflammation. This period typically occurs within the first 24 h of injury and lasts up to 2 days. Additionally, activation of immune cells within the tissue may occur. Inflammatory cells are important to wound healing as they contribute to releasing reactive oxygen species and lysosomal enzymes and removing cell debris. Within 48 h of injury, monocytes migration from neighbouring blood vessels intensifies and are subsequently differentiated into macrophages. These macrophages release growth factors which trigger tissue formation at the injury site. Towards the end of the inflammatory phase, the proliferation phase initiates to diminish the injured tissue area via contraction and fibroplasia. This creates a suitable environment for the subsequent activation of keratinocytes. This phase occurs within 48 h and continues for up to 14 days after the initial injury. Vascular remodelling occurs through the production of collateral veins and facilitates the transport of fluids and nutrients to the stroma. Granulation tissue begins to form within 4 days of the injury. This tissue aids in wound healing by filling the wound bed, providing scaffolding for later stages of healing. Granulation tissue also plays a role in synthesising and depositing additional extracellular components such as collagen, proteoglycans, and fibronectin. These components improve the integrity of the healing tissue. The resulting scaffolding of granulation tissue then provides a suitable substrate for the proliferation of epithelial cells. The final stage of wound healing, the remodelling stage, aims to maximise the tensile strength of the extracellular matrix. This process begins within 2–3 weeks of injury and can last a year or longer. Collagen remodelling takes place, and the initially deposited collagen fibres that are highly disorganised become realigned along the lines of tension. A balance of collagen synthesis and degradation occurs to achieve a more organised collagen matrix. Scar maturation occurs when scar tissue formed during the healing stage changes. These changes result in a flatter scar, which is closer in appearance to the surrounding healthy tissue. Fibroblasts, which initially produce collagen, differentiate into fibrocytes, which play a role in maintaining tissue integrity. The outcome of the three phases is a healed wound with improved tissue strength and function.^40^

**TABLE 1:** Antimicrobial and anti‐inflammatory mechanisms of action of nanocrystalline silver.

Antimicrobial mechanism	Anti‐inflammatory mechanism
Interrupts enzymes and components associated with cellular respiration	Modulates the release of cytokines, modulates oxidative stress, and suppresses NF‐κβ signalling
Impairs DNA functions and alters cellular membrane permeability	Inhibits inflammatory mediators
Disrupts biofilms	Regulates immune cells

In addition to potent antimicrobial, antifungal, and antiviral properties, nanocrystalline silver exhibits anti‐inflammatory effects.^41^ The mechanisms that result in the modulation of inflammation are still under investigation; however, multiple mechanisms have been identified. Silver controls inflammation by modulating the release of cytokines, inhibiting inflammatory mediators, modulating oxidative stress, suppressing NF‐κB signalling, and regulating immune cells.^42^ Additionally, nanomolecular silver may interrupt the signalling pathway of inflammation.^41^ An important note is that the specific mechanisms by which nanocrystalline silver modulates inflammation are still under investigation. The anti‐inflammatory properties of nanocrystalline silver dressings have been examined using a porcine model of contact dermatitis.^43^ Following the induction of inflammation with dinitrochlorobenzene and daily treatment with nanocrystalline silver dressings, erythema, oedema, and histological data were collected. The nanocrystalline silver treatment group showed decreased inflammation due to decreased expression of proinflammatory cytokines, increased inflammatory cell apoptosis, and decreased gelatinase activity. This study supported the hypothesis that Ag^0^, unique to nanocrystalline silver, may regulate inflammation.

Although inflammation is critical to wound healing, excessive inflammation can harm the healing process. For example, excessive inflammation can delay healing, increase tissue damage, increase infection risk, and result in excessive scarring and exacerbate chronic wounds.^44^ Conversely, reducing inflammation results in the formation of less scar tissue. To facilitate optimal wound treatment, it thus becomes necessary to regulate the inflammatory response. Inflammation is an important part of the body's immune response because it protects against infection, aids in tissue repair, prevents excessive tissue damage and helps to restore homeostasis.^45^ Inflammation occurs due to injury where the host's immune cells infiltrate the affected tissue. Restoring tissue homeostasis includes the restoration of the cell number and composition, reconstruction of tissue architecture, restoring structural components such as cell junctions and the basement membrane, adjusting concentrations of metabolic products, oxygen, and nutrients, adjusting pH and temperature, and restoring osmolarity of interstitial fluids. Following injury, specialised sensors detect changes to regulated variables, including cell volume, pH, reactive oxygen species, and electrolyte concentrations. These sensors incorporate both sensory neurons and endocrine cells. The sensors of tissue homeostasis that detect the extreme changes associated with injury and infection include tissue‐resident immune cells, primarily macrophages and mast cells, and somatosensory neurons, for example, C‐fibre nociceptors. When these sensors detect changes, tissue‐autonomous defences activate; when these functions are insufficient, an acute inflammatory response occurs. Basophils, neutrophils, eosinophils, and monocytes facilitate this response. In addition to inflammation occurring following extreme changes to homeostasis, it may also onset due to disruption by agents. These agents include pathogens, xenobiotics, and toxins. When changes or disruption occurs, direct recognition is used to elicit the inflammatory response before tissue damage occurs. Another recognition mechanism does not rely on directly sensing the disruption to homeostasis. These indirect mechanisms restore homeostasis by recognising changes to functional features, including enzymatic activity and membrane pore formation. This indirect mechanism is primarily used to restore homeostasis following disruption by toxins and poisons.^46^ Microbial structures called pathogen‐associated molecular patterns (PAMPS) can trigger an inflammatory response by activating germline‐encoded pattern‐recognition receptors (PRRs), which are expressed in immune and nonimmune cells.^47^ Classes of PRR include Toll‐like receptors (TLRs), which, when activated, activate an intracellular signalling cascade. This signalling cascade leads to the nuclear translocation of transcription factors, including activator protein‐1 and NF‐κB. The intracellular signalling results in the active production of inflammatory mediators. These primary inflammatory stimuli consist of tumour necrosis factor‐⍺ (TNF‐⍺), interleukin‐1β (IL‐1β), and interleukin‐6 (IL‐6). The NF‐κB pathway is important in inflammatory, immune response, and apoptosis. This pathway regulates pro‐inflammatory cytokine production in addition to inflammatory cell recruitment. The MAPK pathway comprises serine/threonine protein kinases that direct cellular responses. Activation of this pathway results in phosphorylation and activation of p38 transcription factors, initiating the inflammatory response. The JAK–STAT pathway comprises various cytokines, interferons, growth hormones, and leptin. Signalling of this pathway results in extracellular factors that adjust gene expression. These three pathways, the NF‐κB, MAPK, and JAK–STAT pathways, play a key role in inflammation; subsequently, deregulation of any of these pathways may result in inflammatory‐associated diseases.

This section discussed multiple mechanisms of action regarding nanocrystalline silver's antimicrobial and anti‐inflammatory properties. These mechanisms of action have been supported by discussing important details as to inflammatory pathways and the restoration of homeostasis, as well as how excessive inflammation results in decreased wound healing.

### 3.4 Clinical effectiveness

Table 2 provides an overview of the critical clinical studies that demonstrate the clinical indications of the use of nanocrystalline silver dressings. Present‐day uses of nanocrystalline silver dressings result in expedited healing, compared to topical applications of SSD, and are not subject to many of the limitations of the surgical approach.^48^ Unlike silver nitrate or SSD, nanocrystalline silver dressings release silver at lower concentrations than other silver‐based modalities; nanocrystalline silver dressings release multiple species of silver, which reduces the probability of resistance developing.^49^ In addition to antimicrobial properties, nanocrystalline silver‐containing dressings exhibit anti‐inflammatory properties due to the delivery mechanism of the nanocrystalline silver. Nanocrystalline silver delivery results in the modulation of inflammation at or above the TNF‐⍺ level, and it includes apoptosis, which prevents cells from becoming necrotic. In a small clinical trial of 10 patients conducted in Calgary, Canada, the effects of nanocrystalline silver's anti‐inflammatory properties were evaluated. One of the trial subjects, an 86‐year‐old female with a 10‐year history of venous ulcers, had been assessed daily and treated with 1% silver sulphadiazine cream and pressure dressings for the previous 2 years. At first, only the right foot was used in the study and treated with nanocrystalline silver, but when it was observed that the persistent wounds were healing, the left foot was included as well. Inflammatory markers MMP‐9 and MMP‐2 were evaluated throughout the duration of the study. Following the last application of silver sulphadiazine to the first application of silver dressings, MMP‐2 decreased, and MMP‐9 levels decreased dramatically. Drug‐resistant infections are increasingly concerning; silver resistance has rarely been observed or reported. Furthermore, nanocrystalline silver dressings are unlikely to result in resistance as the dressings release multiple species of silver at lower concentrations, unlike other silver agents that release higher concentrations of only Ag^+^ ions (including silver nitrate and silver sulphadiazine). Additionally, the multiple mechanisms of action of silver contribute to silver's rarely observed resistance. For resistance to occur, multiple simultaneous mutations of an organism would need to occur for each mechanism of action before the given organism would develop resistance. Table 2 provides an overview of additional important clinical trials and their conclusions.

**TABLE 2:** Significant studies on the clinical effectiveness of nanocrystalline silver.

Study	Year	Conclusions	Reference
Infection and the chronic wound: a focus on silver	2005	Nanocrystalline silver dressings significantly reduce persistent venous ulcers and inflammatory markers MMP‐9 and MMP‐2 in a patient with chronic wounds, highlighting silver's anti‐inflammatory effects.	Warriner et al.^49^
A retrospective review of the use of a nanocrystalline silver dressing in the management of open chronic wounds in the community	2021	Chronic wounds treated with silver dressings substantially reduce healing time, with improvements of up to 50% in wound closure rates.	Hurd et al.^50^
The safety and efficacy of dressings with silver—addressing clinical concerns	2007	The use of Acticoat™ significantly reduces the incidence of local wound infections and systemic bacteremia in chronic wound care.	Cutting et al.^51^
Screening evaluation of an ionised nanocrystalline silver dressing in chronic wound care	2001	Given its broad‐spectrum antimicrobial properties, silver is highly effective for chronic wounds with mixed bacterial populations. It notably reduces wound microflora.	Sibbald et al.^52^
The rate of re‐epithelialization across meshed skin grafts is increased with exposure to silver	2002	Silver‐based dressings like Acticoat™ improve epithelialization times in grafted burn wounds by approximately 40% over non‐silver dressings.	Demling et al.^53^
A matched‐pair, randomised study evaluating the efficacy and safety of Acticoat silver‐coated dressing for the treatment of burn wounds	1998	Silver nanocrystal dressings are preventative against infection in burn patients, offering a promising alternative to traditional silver nitrate treatments.	Tredget et al.^54^

### 3.5 Considerations of use

In addition to the effectiveness of a given treatment, there are many other considerations of use. For example, wounds and burns can vary in size, severity, and potential susceptibility to infection. Given this, treatments need to be selected based on the specific injury. Other factors that factor into treatments being considered include acknowledging potential use challenges. For silver dressings, these potential challenges that may be considered can consist of concerns regarding cytotoxicity, allergic reactions, development of silver resistance, cost‐effectiveness, and availability. As addressed previously, concerns regarding nanocrystalline silvers' cytotoxicity and resistance are largely unsubstantiated. However, concerns regarding cost and availability may be more likely to factor into decision‐making.

As of 2013, the market for advanced wound care products was predicted to be approximately $4.4 billion, and the field continues to grow.^55^ These costs do not include those associated with hospitalizations, physicians, or diagnostic fees. For example, treating a diabetic foot ulcer starts at approximately $9000. When diabetic foot ulcers become infected, the price of treatment rises to approximately $17,000 when the infection is treatable and $45,000 when the infection leads to amputation. The cost of nanocrystalline silver dressings must be compared to the associated treatment costs, especially for wounds that lead to infection. Considering the patient's quality of life and the increased cost of treatment when a wound becomes infected, the use of nanocrystalline silver dressings should be seriously considered despite being more costly than some treatment modalities.

## 4 CONCLUSION

Silver has been recognised as an effective antimicrobial agent and has thus been used throughout history in different forms for wound care. This literature review explored the historical context and current uses of silver dressings for wounds and burns. In addition, this review studied the evolution of silver dressings, their mechanisms of action, clinical effectiveness, and potential indications and contraindications. The findings demonstrated that silver dressings significantly benefit wound healing and infection control, making them an important treatment modality in contemporary wound care management. This review explored the existing approaches to wound care, historical uses of silver, nanocrystalline silver structure and properties, the mechanisms of action of silver that result in its antimicrobial and anti‐inflammatory properties, examples of silver dressings, the clinical effectiveness of silver‐based dressings, indications and contraindications of use, and future directions and innovations of silver dressings.

Future research on nanocrystalline silver for wound care will likely focus on refining nanoparticle size, stability, and delivery mechanisms to improve efficacy whilst minimising toxicity. Additionally, further clinical trials and in vivo studies will be necessary for nanocrystalline silver dressings to be used in a larger capacity. Current research gaps include understanding the anti‐inflammatory mechanisms of nanocrystalline silver, the long‐term effects of silver accumulation in tissue, and the development of delivery systems that release silver selectively in response to infection markers, such as pH and temperature changes in the wound microenvironment. Clinically, expanding studies on patient‐specific factors and wound types may guide the application of nanocrystalline silver dressings and enhance patient outcomes. However, barriers to broader use persist, including the high cost of nanocrystalline silver dressings. As the field progresses, innovations that address this challenge may establish nanocrystalline silver as a standard treatment in complex wound care management.

## CONFLICT OF INTEREST STATEMENT

The authors declare no conflicts of interest.

## Data Availability

Data sharing is not applicable to this article as no new data were created or analyzed in this study.
